# Ellagitannins and Oligomeric Proanthocyanidins of Three Polygonaceous Plants

**DOI:** 10.3390/molecules26020337

**Published:** 2021-01-11

**Authors:** Yun-Qiu Li, Masako Kitaoka, Juri Takayoshi, Ya-Feng Wang, Yosuke Matsuo, Yoshinori Saito, Yong-Lin Huang, Dian-Peng Li, Gen-ichiro Nonaka, Zhi-Hong Jiang, Takashi Tanaka

**Affiliations:** 1 Department of Natural Product Chemistry, Graduate School of Biomedical Sciences, Nagasaki University, 1-14 Bunkyo-machi, Nagasaki 852-8521, Japan; leeyq88@126.com (Y.-Q.L.); y-matsuo@nagasaki-u.ac.jp (Y.M.); saiyoshi@nagasaki-u.ac.jp (Y.S.); 2 College of Medical Laboratory Science, Guilin Medical University, 109, Huancheng North 2 Road, Guilin 541004, China; 3 Department of Natural Product Chemistry, School of Pharmaceutical Sciences, Nagasaki University, 1-14 Bunkyo-machi, Nagasaki 852-8521, Japan; bb00509015@cc.nagasaki-u.ac.jp (M.K.); bb30217021@ms.nagasaki-u.ac.jp (J.T.); 4 Guangxi Key Laboratory of Functional Phytochemicals Research and Utilization, Guangxi Institute of Botany, Guilin 541006, China; wyf@gxib.cn (Y.-F.W.); hyl@gxib.cn (Y.-L.H.); ldp@gxib.cn (D.-P.L.); 5 Usaien Pharmaceutical Company, Ltd., 1-4-6 Zaimoku, Saga 840-0055, Japan; usaien@lily.ocn.ne.jp; 6 Institute for Applied Research in Medicine and Health, Macau University of Science and Technology, Taipa, Macau 999078, China; zhjiang@must.edu.mo

**Keywords:** ellagitannin, hydrolyzable tannin, proanthocyanidin, Persicaria, Polygonum, Polygonaceae

## Abstract

The aim of this study was to characterize hydrolyzable tannins in Polygonaceous plants, as only a few plants have previously been reported to contain ellagitannins. From *Persicaria chinensis*, a new hydrolyzable tannin called persicarianin was isolated and characterized to be 3-*O*-galloyl-4,6-(*S*)-dehydrohexahydroxydiphenoyl-d-glucose. Interestingly, acid hydrolysis of this compound afforded ellagic acid, despite the absence of a hexahydroxydiphenoyl group. From the rhizome of *Polygonum runcinatum* var. *sinense*, a large amount of granatin A, along with minor ellagitannins, helioscpoinin A, davicratinic acids B and C, and a new ellagitannin called polygonanin A, were isolated. Based on 2D nuclear magnetic resonance (NMR) spectroscopic examination, the structure of polygonanin A was determined to be 1,6-(*S*)-hexahydroxydiphenoyl-2,4-hydroxychebuloyl-β-d-glucopyranose. These are the second and third hydrolyzable tannins isolated from Polygonaceous plants. In addition, oligomeric proanthocyanidins of *Persicaria capitatum* and *P. chinensis* were characterized by thiol degradation. These results suggested that some Polygonaceous plants are the source of hydrolyzable tannins not only proanthocyanidins.

## 1. Introduction

**Figure 1:**
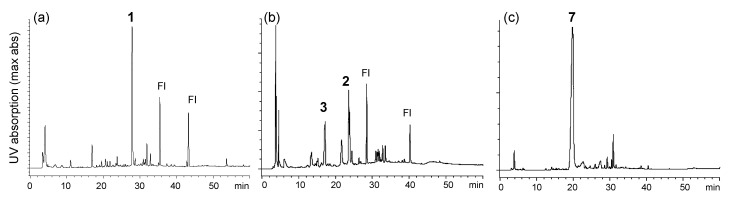
HPLC profiles of 60% CH_3_CN extracts of (**a**) *Persicaria capitata*, (**b**) *Persicaria chinensis* and (**c**) *Polygonum runcinatum* var. *sinense.* **1**: davidiin, **2**: geraniin, **3**: persicarianin, **7**: granatin A and Fl: flavonoid glycosides.

**Figure 2:**
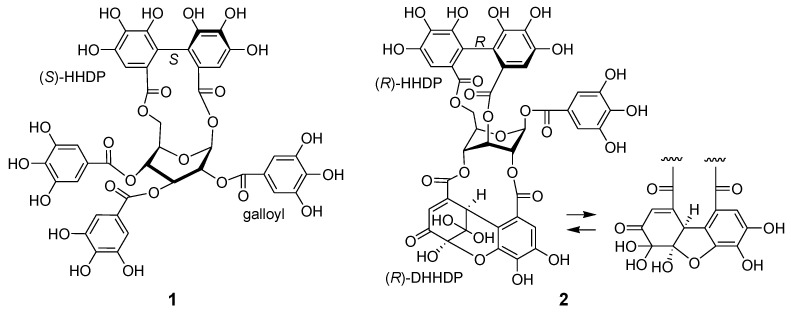
Structures of davidiin (**1**) from *Persicaria capitata* and geraniin (**2**) from *Persicaria chinensis.*

Ellagitannins are hydrolyzable tannins containing hexahydroxydiphenoyl (HHDP) groups and related acyl groups. Recently, their high structural diversity and biological activities have attracted intense interest amongst natural product chemists [1,2,3,4,5]. Compared to proanthocyanidins (synonym (syn.) condensed tannins), distribution of ellagitannins in the plant kingdom is relatively limited. Among Polygonaceous plants, only 2,3,4-tri-*O*-galloyl-1,6-(*S*)-HHDP-β-d-glucose (davidiin, **1**) has been isolated from *Persicaria capitatum* (syn. *Polygonum capitatum*) [6]; its biological activity and metabolism have also been reported [7,8,9]. More recently, the presence of geraniin and chebulagic acid in the aerial parts of *Persicaria chinense* var. *hispidum* was suggested by the use of high-performance liquid chromatography (HPLC)-mass spectroscopy (MS) [10]. In the course of chemical studies on ellagitannins, we analyzed aqueous CH_3_CN extracts of eight Polygonaceous plants, *Reynoutria japonica*, *Persicaria perfoliata*, *P. longiseta*, *P. lapathifolia*, *P. capitata*, *P. chinensis*, *P. filiformis*, and *P. thunbergii*, by HPLC-diode array detector (DAD) analysis (Figure S1, Supplementary Materials). In the study, only the extracts of *P. capitata* and *P. chinensis* showed peaks with UV absorption characteristic to ellagitannins (Figure 1). The results were consistent with the above-mentioned studies; the major ellagitannin of *P. capitata* was davidiin (**1**), and *P. chinensis* contains geraniin (**2**) (Figure 2). In addition, we found that the rhizome of *Polygonum runcinatum* var. *sinense*, a Chinese perennial plant used as a folk medicine for detoxification and hemostasis, contains ellagitannins as the major constituents. This paper describes the isolation of phenolic substances from these three plants and structural determination of the two new ellagitannins obtained from *Persicaria chinensis* and *Polygonum runcinatum* var. *sinense.* In addition, proanthocyanidin oligomers from *Persicaria capitata* and *Persicaria chinensis* were chemically characterized.

## 2. Results and Discussion

### 2.1. Polyphenols of Persicaria capitata

The EtOH extract of *Persicaria capitata* whole plant, collected in Shui Cheng, Guizhou, China, was fractionated by Diaion HP20SS and further separated using Sephadex LH-20 and Chromatorex ODS to give davidiin (**1**) as the major constituent (7% from the extract), along with minor compounds, which were identified as 2,3-di- [11], 2,4-di- [12], 2,3,4-tri- [13], and 2,3,6-tri-*O*-galloyl glucoses [14], 6’-*O*-galloyl arbutin [15], 4-hydroxy-3-methoxyphenol 1-*O*-β-d-(6’-*O*-galloyl)-glucopyranoside [16], 4-hydroxy-2-methoxyphenol 1-*O*-β-d-(6’-*O*-galloyl)-glucopyranoside [16], quercetin 3-*O*-β-d-glucoside [17], quercetin 3-*O*-α-L-rhamnopyranoside [17], quercetin 3-*O*-α-L-(3’’-*O*-galloyl)-rhamnopyranoside [18], ellagic acid, and quercetin.

### 2.2. Polyphenols from Persicaria Chinensis

**Figure 3:**
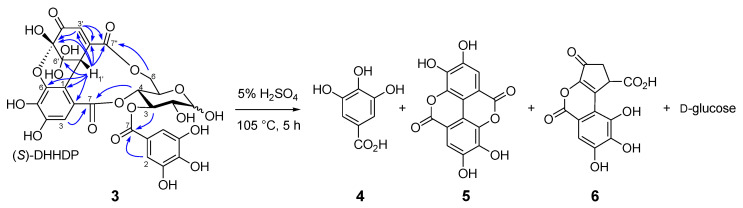
Selected heteronuclear multiple bond coherence (HMBC) correlations and acid hydrolysis of 3-*O*-galloyl-4,6-(*S*)-DHHDP-d-glucose (**3**).

Similar separation of an aqueous acetone extract of the fresh aerial part of *Persicaria chinensis* collected in Nagasaki, Japan, afforded 1-*O*- [19] and 1,2,6-tri-*O*-galloyl-β-d-glucoses [20], geraniin (**2**) [21], quercetin 3-*O*-(2’’-α-rhamnopyranosyl)-β-glucuronopyranoside [22], and a new ellagitannin called persicarianin. Persicarianin (**3**) was obtained as a brown amorphous powder, and high-resolution electrospray ionization time of flight (HRESITOF) MS indicated a molecular formula of C_27_H_22_O_19_ (*m/z*: 673.0689, calculated for C_27_H_22_O_19_Na: 673.0653). The ^1^H NMR spectrum showed signals arising from α- and β-hexopyranoses, and it is apparent that the sugar is a ^4^C_1_-glucopyranose based on the large coupling constants of the pyranose ring proton signals (*J*_2,3_, *J*_3,4_, *J*_4,5_ = 8–10 Hz). The low field shifts of H-3 [δ 5.63 (α-H-3), 5.40 (β-H-3)], H-4 [5.40 (α-H-4), 3.94 (β-H-4)], and H-6 [4.94, 3.95 (α-H-6), 5.40, 3.92 (β-H-6)] indicates acylation of these positions. The acyl groups were shown to be a galloyl and a dehydrohexahydroxydiphenoyl (DHHDP) group by ^13^C NMR spectroscopy, which exhibited signals attributable to an aliphatic methine (δ 43.0), a double bond (δ 151.7 and 130.8), a ketone (δ 192.0), and two acetal carbons (δ 91.5 and 96.5), constructing a hydrated cyclohexenetrione ring of the DHHDP group. The large chemical shift differences of the glucose H-6 methylene proton signals in the ^1^H NMR spectrum suggested that the DHHDP esters bridges between the C-4 and C-6 hydroxy groups [23]. This was confirmed by heteronuclear multiple bond coherence (HMBC) correlations of the glucose H-6 and H-4 to the DHHDP C-7’ and C-7 ester carbonyl carbons, respectively (Figure 3). The configuration of the DHHDP methine carbon (C-1’) was concluded to be *S,* based on the appearance of negative and positive Cotton effects at 213 nm and 234 nm, respectively [24]. The location of the galloyl group was determined to be at the glucose C-3 hydroxy group by observation of HMBC correlations between the glucose H-3 and galloyl C-7. The D configuration of the glucosyl moiety was determined via acid hydrolysis followed by HPLC analysis of the thiazolidine derivatives prepared by reaction with L-cysteine and *o*-tolylisothiocyanate [25,26]. Based on this evidence, persicarianin was characterized to be 3-*O*-galloyl-4,6-(*S*)-DHHDP-d-glucose (**3**). HPLC analysis of the aforementioned hydrolysis products, before condensation with cysteine, revealed production of gallic acid (**4**), ellagic acid (**5**), and brevifolin carboxylic acid (**6**) (Figure S2, Supplementary Materials). Ellagic acid is a bislactone form of the HHDP group, and therefore, a reduction product of the DHHDP ester of **3**. Similar production of **5** from DHHDP groups on acid hydrolysis have been also observed for **2** and related ellagitannins, and the reaction was deduced to be a redox disproportionation [27,28]. Ellagitannins were originally defined as hydrolyzable tannins which afford **5** upon hydrolysis, and **5** is usually considered to be originated from HHDP groups. In this context, despite the absence of a HHDP group, **3** is also an ellagitannin.

### 2.3. Hydrolyzable Tannin from Polygonum Runcinatum var. Sinense

**Figure 4:**
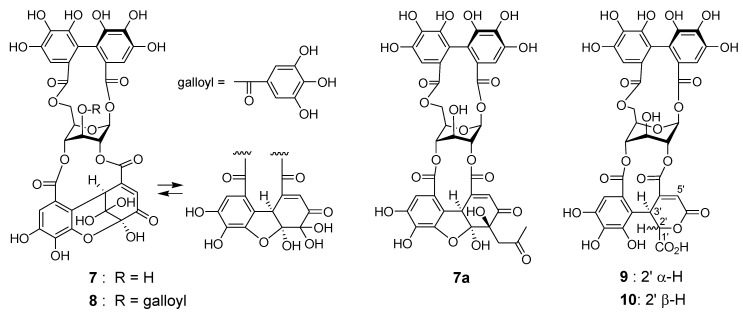
Structures of **7–10** and acetonyl derivative **7a**.

Reverse-phase HPLC analysis of the rhizome of *Polygonum runcinatum* var. *sinense* showed a prominent peak arising from a principal phenolic component (Figure 1c), which was isolated by Diaion HP20SS column chromatography and identified as granatin A (**7**) by 1D and 2D NMR spectroscopic analysis [29]. Furthermore, the acetonyl derivative **7a** was prepared by treatment of **7** with aqueous acetone containing HCO_2_NH_4_, and spectroscopic comparison of **7a** with those of an authentic sample provided further evidence for the structure of **7** [30]. In the purification process of **7** (Figure 4), four minor ellagitannins were isolated by a combination of column chromatography using Chromatorex ODS and Toyopearl Butyl-650M to give helioscopinin A (**8**) [31], davicratinic acids B (**9**) and C (**10**) [32], and a new ellagitannin called polygonanin A.

**Figure 5:**
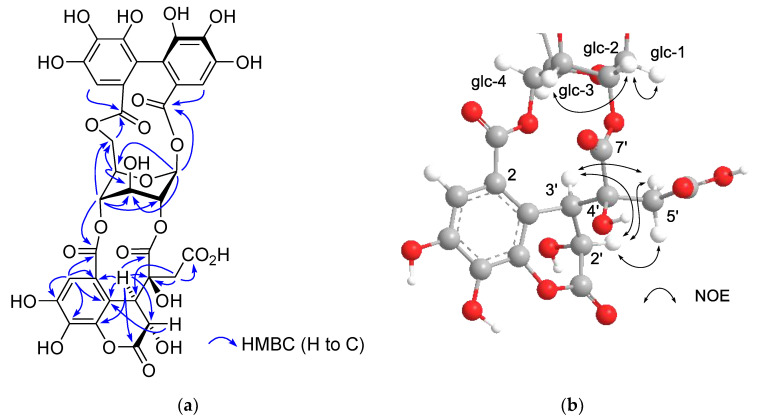
(**a**) Structure and selected HMBC correlations for **11**. (**b**) A partial structure of the most stable conformer of **11** and nuclear Overhauser effect spectroscopy (NOESY) correlations of the glucose and 2,4-acyl group.

**Scheme 1:**
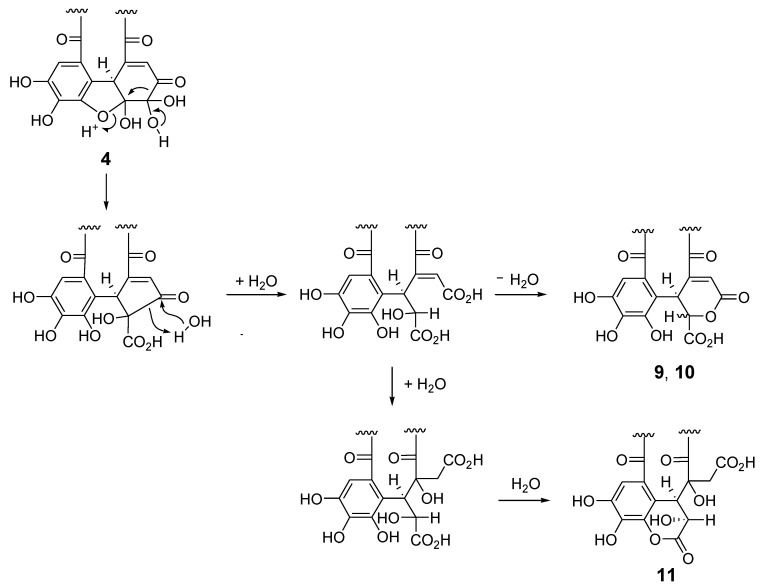
Plausible production mechanism of **9–11** from **4**.

The ^1^H and ^13^C NMR spectra of polygonanin A (**11**) was related to those of **9** and **10**, indicating 1,2,4,6-acylated glucopyranose with a HHDP group. The location of the HHDP group at the 1,6-positions of glucose was confirmed by HMBC correlations of glucose H-1 (δ_H_ 5.95, s) and H-6 [δ_H_ 4.99 (t, *J* = 11.1 Hz), 4.08 (dd, *J* = 5.3, 11.1 Hz)] with the HHDP ester carbonyl carbons (δ_C_ 166.4, 168.2). The atropisomerism of the HHDP was shown to be an *S* configuration by appearance of positive and negative Cotton effects at 242 nm and 262 nm, respectively, in the electronic circular dichroism (ECD) spectrum. The configuration is the same as that of **1**, **7**, **9,** and **10**. Coupling constants of the pyranose ring protons (*J*_1,2_, *J*_2,3_, *J*_3,4_, and *J*_4,5_) were <2 Hz. This was similar to those of **7, 9**, and **10**, but different to those of **1** (*J*_1,2_ = 3.2 Hz, *J*_2,3_ = 8.5 Hz, *J*_3,4_ = 7.7 Hz, and *J*_4,5_ = 2.9 Hz), suggesting that the glucopyranoses of **7**, **9**, **10**, and **11** adopt a ^1^C_4_ conformation, whereas the glucopyranose core of **1** with a 2,3,4-trigalloyl structure adopts a boat conformation (Figure 1). The NMR signals indicated that the 2,4-acyl group of **11** was composed of 3 carboxyl carbons (δ_C_ 172.3 (C-1’), 172.0 (C-6’), 166.1 (C-7’)), an oxygenated tertiary carbon (δ_C_ 76.3 (C-4’)), an oxygenated methine (δ_C_ 66.3 (C-2’)), a methylene (δ_C_ 41.6 (C-5’)), and a benzylic methine (δ_C_ 49.2 (C-3’)), along with a trihydroxy benzoyl moiety. Taking the molecular formula C_34_H_26_O_24_ indicated by HR-fast atom bombardment (FAB) MS into account, these building blocks of the 2,4-acyl group suggested that **8** is generated by addition of H_2_O to the double bond of **9** or **10** and rearrangements of lactone formation. This was supported by HMBC correlations, as illustrated in Figure 5. The planar structure of this acyl group is the same as the 4’-hydroxychebuloyl group of an ellagitannin isolated from a Euphorbiaceous plant [33], and the chemical shifts of the aliphatic proton and carbon signals in the literature (δ_H_ 4.84 (d, *J* = 5 Hz, H-2’), 4.90 (d, *J* = 5 Hz, H-3’), 3.28 and 3.43 (each d, *J* = 18 Hz, H-5’); δ_C_ 68.5 (C-2’), 47.3 (C-3’), 77.6 (C-4’), 42.5 (C-5’)) are similar to those of **11** (δ_H_ 4.83 (br s, HChe H-2’) and 4.88 (d, *J* = 2.8 Hz, HChe H-3’), 3.35 and 2.78 (each d, *J* = 15.9 Hz, HChe H-5’); δ_C_ 66.3 (C-2’), 49.2 (C-3’), 76.3 (C-4’), 41.6 (C-5’)). The configuration of C-2’, C-3’, and C-4’ was concluded to be S*, R*, and S* based on the nuclear Overhauser effect spectroscopy (NOESY) correlations between the H-2’, H-3’ and H-5’ of **11**. The most stable conformation of **11** obtained by computational calculation along with NOESY correlations is shown in Figure 5b. The NOESY correlation between H-2’ and one of the H-5’ methylene protons confirmed the relative configurations of C-2’ – C-5’. From the biogenesis illustrated Scheme 1, the configuration of the benzylic methines of the acyl groups of **11** was deduced to be the same as that of the DHHDP group of **7**. Interestingly, the glucose H-3 of **11** (δ_H_ 5.14) resonated at a much lower field compared to those of **7a** (δ_H_ 4.58), **9** (δ_H_ 4.40) and **10** (δ_H_ 4.44). This could be due to the deshielding effect of the ester carbonyl group attached to the glucose C-2 hydroxy group (Figure 5b), suggesting that hydroxylation at C-4’ of the 2,4-acyl group affects the conformation of the macrocyclic ester structure.

### 2.4. Proanthocyanidins of Persicaria Capitata and Persicaria Chinensis

**Figure 6:**
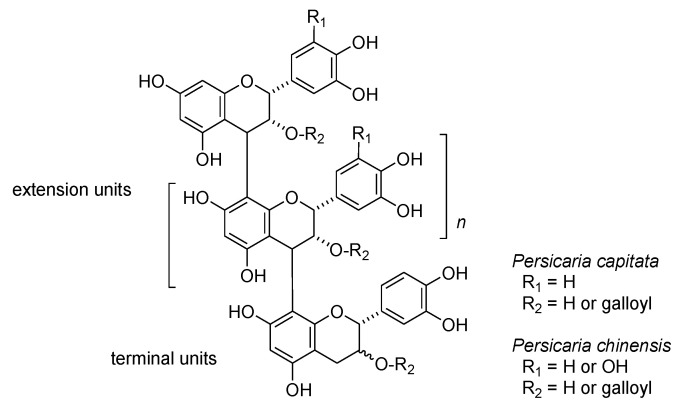
Structures of oligomeric proanthocyanidins of *Persicaria capitata* and *P. chinensis* suggested by thiol degradation.

In addition to hydrolyzable tannins, oligomeric proanthocyanidins are also important constituents of *Persicaria capitata* and *Persicaria chinensis*. The oligomers were detected as broad humps on the HPLC baseline; thiol degradation using 2-mercaproethanol was used to characterize the structural components [34] (Figure S3, Supplementary Materials). HPLC analysis of the degradation products obtained from proanthocyanidins of *Persicaria capitata* exhibited peaks attributable to 4β-(2-hydroxyethylsulfanyl) derivatives of epicatechin and epicatechin-3-*O*-gallate originating from extension units, accompanied by small peaks of catechin, epicatechin, and epicatechin gallate arising from terminal units. Oligomeric proanthocyanidins of *P. chinensis* yielded 2-hydroxyethylsulfanyl derivatives of epicatechin, epigallocatechin, epicatechin-3-*O*-gallate and epigallocatechin-3-*O*-gallate originating from extension units, along with the free form of epicatechin-3-*O*-gallate originating from the terminal unit. The results indicates that proanthocyanidins in these two plants belonging the same genus *Persicaria* are composed of different flavan-3-ol units (Figure 6). In contrast, high-molecular weight polyphenols obtained from the rhizome of *Polygonum runcinatum* var. *sinense* did not yield 2-hydroxyethylsulfanyl derivatives on thiol degradation, indicating that the polyphenols are not proanthocyanidins. The ^13^C NMR spectrum suggested that the polyphenols are oligomeric hydrolyzable tannins. Further investigations are now underway.

## 3. Materials and Methods

### 3.1. General Information

Optical rotations were measured on a JASCO P-1020 digital polarimeter (JASCO, Tokyo, Japan). IR spectra were measured on a JASCO FT/IR 410 spectrophotometer. Ultraviolet (UV) spectra were obtained on a JASCO V-560 UV/VIS spectrophotometer. ECD spectra were measured with a JASCO J-725N spectrophotometer. ^1^H- and ^13^C-NMR spectra were recorded on a Varian Unity plus 500 spectrometer (Agilent Technologies, Santa Clara, CA, USA) operating at 500 MHz and 126 MHz for the ^1^H and ^13^C nuclei, respectively. ^1^H- and ^13^C-NMR spectra were also recorded on a JEOL JNM-AL400 spectrometer (JEOL Ltd., Tokyo, Japan) operating at 400 and 100 MHz for the ^1^H and ^13^C nuclei, respectively. Coupling constants (*J*) were expressed in hertz and chemical shifts (δ) are reported in ppm with the solvent signal used as a standard (pyridine-*d*_5_: δ_H_ 7.19, δ_C_ 123.5 and methanol-*d*_4_: δ_H_ 3.31, δ_C_ 49.0). FAB-MS data were recorded on a JMS700N spectrometer (JEOL Ltd., Tokyo, Japan) using *m*-nitrobenzyl alcohol or glycerol as the matrix. ESI-TOF-MS data were recorded on a quadrupole (Q)-TOF LC/MS (Agilent 6550, Agilent Technologies, Santa Clara, CA, USA). Column chromatography was performed using Sephadex LH-20 (25–100 μm, GE Healthcare UK Ltd., Little Chalfont, UK), Diaion HP20PSS (Mitsubishi Chemical Co., Tokyo, Japan), Chromatorex ODS (Fuji Silysia Chemical Ltd., Kasugai, Japan), and Toyopearl butyl-650M (Tosoh Corporation, Tokyo, Japan) columns. TLC was performed on precoated Kieselgel 60 F254 plates (0.2 mm thick, Merck, Darmstadt, Germany) with CHCl_3_-MeOH-H_2_O (10:3:0.5 or 7:3:0.5, *v/v*) and toluene-ethyl formate-formic acid (1:7:1, *v/v*) mixtures being used as the eluents. The spots were detected using UV illumination and by spraying with a 5% H_2_SO_4_ solution followed by heating. Analytical HPLC was performed on a Cosmosil 5C18-ARII (Nacalai Tesque, Kyoto, Japan) column (250 mm × 4.6 mm, i.d.) with a gradient elution of 4–30% (39 min) and 30–75% (15 min) CH_3_CN in 50 mM H_3_PO_4_ at 35 °C (flow rate, 0.8 mL/min; detection, JASCO photodiode array detector MD-2010).

### 3.2. Plant Material

The whole plant of *Persicaria capitata* was collected in Shui Cheng, China, in 2014, and in Nagasaki, Japan, in 2017. The aerial part of *Persicaria chinensis* were collected in Nagasaki, Japan, in 2014. Voucher specimens were deposited at the Nagasaki University Graduate School of Biomedical Sciences. Fresh rhizome of *Polygonum runcinatum* var. *sinense* were purchased in a medicinal plant market in Guangxi, China. A voucher specimen was deposited at the Guangxi Institute of Botany. Aerial parts of *Reynoutria japonica*, *Persicaria perfoliata*, *P. longiseta*, *P. lapathifolia*, *P. filiformis*, and *P. thunbergii* were collected in Nagasaki, Japan.

### 3.3. HPLC Analysis

Fresh aerial parts (1.0 g) of *Reynoutria japonica*, *Persicaria perfoliata*, *P. longiseta*, *P. lapathifolia*, *P. capitata* (syn. *Polygonum capitatum*), *P. chinensis* (syn. *Polygonum chinense*), *P. filiformis*, and *P. thunbergii* were extracted with 60% CH_3_CN (20 mL) and analyzed by HPLC (Figure S1, Supplementary Materials).

### 3.4. Extraction and Separation

#### 3.4.1. Persicaria Capitata

The EtOH extract (200 g) of *P. capitata* was suspended in 50% MeOH and insoluble material was removed by filtration. The soluble part (132 g) was fractionated by Diaion HP20SS column chromatography (8 cm i.d. × 35 cm) with 0–100% MeOH (10% stepwise gradient elution, each 500 mL) to give 8 fractions (fr.): fr. 1 (84.5 g), fr. 2 (2.2 g), fr. 3 (3.9 g), fr. 4 (9.5 g), fr. 1–5 (14 g), fr. 1–6 (4.3 g), fr. 1–7 (6.9 g), and fr. 1–8 (2.1 g). Fr. 3 was subjected to a combination of column chromatography using Sephadex LH-20 (0–100% MeOH), Avicel cellulose (2% AcOH), and Diaion HP20SS (0–50% MeOH) to give 2,3-di-*O*-galloyl-d-glucose (719 mg) and 2,4-di-*O*-galloyl-d-glucose (101 mg). Similar separation of fr. 4 by chromatography using Sephadex LH-20 and Chromatorex ODS (0–50% MeOH, 5% stepwise gradient) to yield 2,3,4-tri-*O*-galloyl-d-glucose (2.26 g), 6-*O*-galloyl arbutin (79 mg), 4-hydroxy-3-methoxyphenol 1-*O*-β-d-(6′-*O*-galloyl)-glucopyranoside (37 mg), 4-hydroxy-2-methoxyphenol 1-*O*-β-d-(6′-*O*-galloyl)-glucopyranoside (46 mg). Fr. 6 was purified by precipitation from water to give davidiin (**1**) (13 g). Fr. 7 was subjected to a column chromatography on Sephadex LH-20 (60–100% MeOH), Diaion HP20SS, and Chromatorex ODS to give quercetin 3-*O*-α-rhamnoside (2.5 g), quercetin (719 mg), quercetin 3-*O*-β-d-glucopyranoside (182 mg), quercetin 3-*O*-α-L-(3”-*O*-galloyl)-rhamnoside (6.7 mg). The insoluble part of the extract (68 g) was washed with CHCl_3_–MeOH (1:1, *v/v*) to remove non-polar substances and a part (5 g) of the residue (31.5 g) was subjected to size-exclusion column chromatography using a Sephadex LH-20 column (4 cm i.d. × 45 cm) with 7 M urea:acetone (2:3, *v/v*, containing conc. HCl at 5 mL/L) to give fractions containing oligomeric polyphenols and compounds with low-molecular weight [35]. The fractions were separated by Diaion HP20SS column chromatography to give oligomeric proanthocyanidins (707 mg) and ellagic acid (54 mg).

#### 3.4.2. Persicaria Chinensis

The fresh aerial part of *P. chinensis* (1.18 kg) was extracted with 60% aqueous acetone (3 L) three times. The extract was concentrated and the resulting insoluble precipitates were removed by filtration. The aqueous filtrate was applied to a column of Diaion HP20SS (7 cm i.d. × 50 cm) with 0–100% MeOH (10% stepwise gradient elution, each 500 mL) to give 11 fractions. Crystallization of fr. 3 from H_2_O yielded 1-*O*-galloyl-β-d-glucopyranose (301 mg). Separation of fr. 7 (4.61 g) by Sephadex LH-20 (0–100% MeOH) gave polymeric proanthocyanidins (1.37 g), **3** (1.37 g), and 1,2,6-tri-*O*-galloyl-β-d-glucopyranose (76 mg). Fr. 8 (11.2 g) was subjected to Sephadex LH-20 (0–100% MeOH and then MeOH–H_2_O–acetone, 8:1:1 and 3:1:1) to give quercetin 3-*O*-(2’’-α-rhamnopyranosyl)-β-glucuronopyranoside (481 mg) and **2** (553 mg).

#### 3.4.3. Polygonum Runcinatum var. Sinense

The dried root of *Polygonum runcinatum* var. *sinense* (805 g) was extracted with 60% acetone (4 L) twice and then MeOH. The extracts were combined and concentrated, and the aqueous solution was applied to a Diaion HP20SS column (8 cm i.d. × 35 cm) with 0–100% MeOH (10% stepwise gradient elution, each 500 mL) to give 13 fractions. HPLC analysis showed that fr. 4–10 (total 159 g) contained **7** as the major component. Fr. 3 (29.7 g) was separated by Sephadex LH-20 column chromatography (6 cm i.d. × 31 cm) with 0–100% MeOH to give **7** (6.68 g), **10** (1.44 g), and a subfraction containing **9** and **11**. Further chromatography of the subfraction using Chromatorex ODS (0–50% MeOH) and Toyopearl butyl-650M (0–50% MeOH) to yield **9** (490 mg) and **11** (19 mg). Fr. 11 (40.2 g) was separated by Sephadex LH-20 (60–100% MeOH), Sephadex LH-20 [7 M urea:acetone (2:3, *v/v*, containing conc. HCl at 5 mL/L)], and Diaion HP20SS column chromatography to afford **8** (990 mg) and a mixture of oligomeric polyphenols detected at origin on TLC plate.

### 3.5. Spectroscopic Data

#### 3.5.1. Persicarianin (**3**)

Brown amorphous powder, [α]_D_ +43.8 (*c* 0.1, MeOH), IR ν_max_ cm^−1^: 3412, 1728, 1708, 1614, 1533, 1450, UV λ_max_ (MeOH) nm (log ε): 271 (4.06), 218 (4.59), HRESIMS *m/z*: 673.0690 (Calculated for C_27_H_22_O_19_Na: 673.0653), ECD (MeOH) Δε (nm): −25.16 (213), +6.68 (234), −5.47 (283), +2.36 (374). ^1^H-NMR (acetone-*d*_6_, 500 MHz) δ_H_: 7.19 (s, α-galloyl-2,6), 7.18 (s, β-galloyl-2,6), 6.76 (s, α,β-DHHDP-6”), 6.38 (s, α-DHHDP-3), 6.37 (s, β-DHHDP-3), 5.63 (dd, *J* = 9.8, 9.5 Hz, α-glc H-3), 5.42 (t, *J* = 9.4 Hz, β-glc H-3), 5.40 (t, *J* = 9.4, α,β-glc H-4), 5.32 (d, *J* = 3.4 Hz, α-glc H-1), 5.01 (dd, *J* = 4.8, 10.7 Hz, α-glc H-6), 4.94 (dd, *J* = 4.8, 10.7 Hz, α-glc H-6), 4.88 (d, *J* = 7.8 Hz, β-glc H-1), 4.65 (s, α-DHHDP-1’), 4.63 (s, β-DHHDP-1’), 4.25 (m, α-glc H-5), 3.95 (m, α-glc H-5, α-glc H-6, β-glc H-6), 3.89 (dd, *J* = 9.8, 3.4 Hz, α-glc H-2), 3.66 (dd, *J* = 9.4, 7.8 Hz, β-glc H-2), ^13^C NMR (acetone-*d*_6_, 125 MHz) δ_C_: 192.0 (α,β-DHHDP-4’), 168.1 (α,β-DHHDP-7), 166.9 (galloyl-7), 164.6 (α,β-DHHDP-7’), 151.7 (α,β-DHHDP-2’), 146.0 (α,β-DHHDP-4), 145.9 (galloyl-3,5), 142.3 (α,β-DHHDP-6), 138.8 (galloyl-4), 135.4 (α,β-DHHDP-5), 130.8 (α,β-DHHDP-3’), 124.1 (α,β-DHHDP-2), 121.7 (galloyl-1), 112.7 (α,β-DHHDP-1), 110.1 (galloyl-2,6), 107.7 (β-DHHDP-3), 107.6 (α-DHHDP-3), 97.8 (β-glc H-1), 96.5 (α,β-DHHDP-5’), 93.6 (α-glc C-1), 91.5 (α,β-DHHDP-6’), 75.6 (β-glc C-3), 74.1 (α-glc C-4), 74.0 (β-glc C-4), 73.7 (β-glc C-2), 73.3 (α-glc C-3), 71.2 (α-glc C-2), 68.3 (β-glc C-5), 66.9 (α-glc C-6), 66.4 (β-glc C-6), 64.4 (α-glc C-5), 43.0 (α,β-DHHDP-1’).

#### 3.5.2. Polygonanin A (**11**)

Tan amorphous powder, [α]_D_ −51.3° (*c* 0.1, MeOH), FABMS *m/z*: 841 (M + Na)^+^, 819 (M + H)^+^, HRFABMS *m/z*: 819.0891 (M + H)^+^ (Calcd for C_34_H_27_O_24_: 819.0887), IR ν_max_ cm^-1^ (neat): 3412, 1719, 1616, 1445, 1313, 1223, 1190. UV λ_max_ (MeOH) nm (log ε): 266 (4.40), 230 (4.70). ECD (MeOH) λ_max_ (Δε): 316 (−2.4), 283 (+12.6), 261 (−8.9), 241 (+17.9), 227 (+0.6), 217 (+5.5). ^1^H NMR (500 MHz, acetone-*d*_6_) δ_H_: 7.46 (1H, s, hydroxychebuloyl (HChe) H-3), 6.83 (1H, s, HHDP-3), 6.79 (1H, s, HHDP-3’), 5.95 (1H, s, glc H-1), 5.14 (1H, br s, glc H-3), 5.10 (1H, br s, glc H-4), 4.99 (1H, t, *J* = 11.1 Hz, glc H-6), 4.88 (1H, d, *J* = 2.8 Hz, HChe H-3’), 4.83 (1H, br s, HChe H-2’), 4.72 (1H, br s, glc H-2), 4.48 (1H, dd, *J* = 5.3, 11.1 Hz, glc H-5), 4.08 (1H, dd, *J* = 5.3, 11.1 Hz, glc H-6), 3.35 (1H, d, *J* = 15.9 Hz, HChe H-5’), 2.78 (1H, d, *J* = 15.9 Hz, HChe H-5’). ^13^C NMR (125 MHz, acetone-*d*_6_) δ_C_: 172.3 (HChe-1’), 172.0 (C-6’), 168.2 (HHDP-7’), 166.7 (HChe-7), 166.4 (HHDP-7), 166.1 (HChe-7’), 146.0 (HChe-4), 145.3 (HHDP-4’), 144.5, 144.9 (HHDP-6, 6’), 143.0 (HChe-6), 144.7 (HHDP-4), 138.5 (HChe-5), 136.9 (HHDP-5), 136.3 (HHDP-5’), 125.9 (HHDP-2), 125.7 (HHDP-2’), 121.0 (HChe-2), 116.7 (HHDP-1), 116.2 (HChe-3), 115.4 (HHDP-1’), 114.5 (HChe-1), 109.8 (HHDP-3), 108.7 (HHDP-3’), 90.4 (glc C-1), 76.3 (HChe-4’), 71.7 (glc C-2), 70.9 (glc C-5), 69.2 (glc C-4), 66.3 (HChe-2’), 64.2 (glc C-6), 59.1 (glc C-3), 49.2 (HChe-3’), 41.6 (HChe-5’).

### 3.6. Acid Hydrolysis of **3**

Persicarianin (**3**) (10 mg) was hydrolyzed by heating in 5% H_2_SO_4_ (1 mL) at 105 °C for 5 h in a screw capped vial. The precipitates (2.7 mg) formed in the mixture were collected by filtration. HPLC analysis of the filtrate showed peaks assignable to gallic acid (8.94 min), brevifolin carboxylic acid (21.36 min), and ellagic acid (30.66 min), which were identified by comparison of *t*_R_ and UV absorption with those of authentic samples. HPLC analysis of the precipitates showed only a peak for ellagic acid. The filtrate was neutralized with saturated Ba(OH)_2_ and resulting precipitate of BaSO_4_ was removed by filtration. The filtrate was concentrated and the residue was dissolved in 0.5 mL of pyridine containing L-cysteine HCl (5.0 mg), and heated at 60 °C for 1 h. To the mixture was added *o*-tolylisothiocyanate (20 μL) and this mixture heated at 60 °C for 1 h. The final mixture was then cooled to ambient temperature and directly analyzed using HPLC. The retention time of the peak at 34.8 min coincided with that of the thiazolidine derivatives of D-glucose (L-glucose: 35. 6 min).

### 3.7. Computational Calculation of **8**

A conformational search was performed using the Monte Carlo method and the MMFF94 force field with Spartan ′14 (Wavefunction, Irvine, CA). The low-energy conformers within a 6 kcal/mol window were optimized at the B3LYP/6-31G(d,p) level in acetone (SMD). The vibrational frequencies were also calculated at the same level to confirm their stability, and no imaginary frequencies were found. The magnetic shielding constants (σ) of the low-energy conformers with Boltzmann populations greater than 1% were calculated using the gauge-independent atomic orbital (GIAO) method at the mPW1PW91/6-311+G(2d,p) level in acetone (PCM) and were weight-averaged [36,37,38].

### 3.8. Thiol Degradation

Polymeric polyphenols obtained from the three plants (5 mg) were dissolved in a solution containing 4% 2-mercaptoethanol and 0.1% HCl in 60% EtOH (1 mL). The mixture was heated at 70 °C for 7 h and analyzed by HPLC. The standard thiol degradation products were obtained from persimmon proanthocyanidins [34].

## 4. Conclusions

Distribution of hydrolyzable tannins in the plant kingdom is much more limited compared with that of proanthocyanidins. In Polygonaceous plants, a hydrolyzable tannin had only been isolated from *Persicaria capitatum*. In this study, we reinvestigated *P. capitatum* and showed the presence of minor gallotannins and proanthocyanidin oligomers comprised of epicatechin and epicatechin-3-*O*-gallate. From *Persicaria chinensis*, a new hydrolyzable tannin called persicarianin (**3**) was isolated together with an ellagitannin geraniin and proanthocyanidin oligomers mainly comprising epicatechin, epigallocatechin and their galloyl esters. The rhizome of *Polygonum runcinatum* var. *sinense* contained granatin A (**7**) as the major constituent along with minor ellagitannins, including a new ellagitannin polygonanin A (**11**). These results prompted us to continue investigations into hydrolyzable tannins in this plant family.

## Supplementary Materials

The following are available online, Figure S1: HPLC of extracts of Polygonaceous plants, Figure S2: HPLC analysis of acid hydrolysis products of **3**, Figure S3: Thiol degradation of proanthocyanidin oligomers, Figures S4–S8: 1D and 2D NMR spectra of **3**, Figures S9–S14: 1D and 2D NMR spectra of **11**. Figures S15 and S16: ^1^H and ^13^C NMR spectra of **7**, Figures S17 and S18: ^1^H and ^13^C NMR spectra of **7a**.

## Sample Availability

Samples are not available from the authors.
