# Supplementary material for: Ellagitannins and Oligomeric Proanthocyanidins of Three Polygonaceous Plants
Source: Molecules. 2021 Jan 11;26(2):337. doi: 10.3390/molecules26020337 (PMC7828057; doi:10.3390/molecules26020337)

Supplementary Materials

Hydrolyzable Tannins and Oligomeric proanthocyanidins of Three Polygonaceous Plants

Yun-Qiu Li [1,2], Masako Kitaoka [1], Juri Takayoshi [1], Ya-Feng Wang [3], Yong-Lin Huang [3,*], Yosuke Matsuo [1], Yoshinori Saito [1], Dian-Peng Li [3], Gen-ichiro Nonaka [4], Zhi-Hong Jiang [5] and Takashi Tanaka [1,*]

[1] Department of Natural Product Chemistry, Graduate School of Biomedical Sciences, Nagasaki University, 1-14 Bunkyo-machi, Nagasaki 852-8521, Japan
[2] College of Medical Laboratory Science, Guilin Medical University, 109, Huancheng north 2 Road, Guilin, Guangxi, 541004, China
[3] Guangxi Key Laboratory of Functional Phytochemicals Research and Utilization, Guangxi Institute of Botany, Guilin 541006, China
[4] Usaien Pharmaceutical Company, Ltd., 1-4-6 Zaimoku, Saga 840-0055, Japan
[5] Institute for Applied Research in Medicine and Health, Macau University of Science and Technology, Taipa, Macau 999078, China
* Correspondence: t-tanaka@nagasaki-u.ac.jp; Tel.: +81-95-819-2432 (T.T.)

Figure S1. HPLC of extracts of Polygonaceous plants.
Figure S2. HPLC analysis of acid hydrolysis products of **3**.
Figure S3. Thiol degradation of proanthocyanidin oligomers.
Figure S4. ^{1}H NMR spectrum of **3**.
Figure S5. ^{13}C NMR spectrum of **3**.
Figure S6. ^{1}H-^{1}H COSY spectrum of **3**.
Figure S7. HSQC spectrum of **3**.
Figure S8. HMBC spectrum of **3**.
Figure S9. ^{1}H NMR spectrum of **11**.
Figure S10. ^{13}C NMR spectrum of **11**.
Figure S11. ^{1}H-^{1}H COSY spectrum of **11**.
Figure S12. HSQC spectrum of **11**.
Figure S13. HMBC spectrum of **11**.
Figure S14. NOESY spectrum of **11**.
Figure S15. ^{1}H NMR spectrum of **7**.
Figure S16. ^{13}C NMR spectrum of **7**.
Figure S17. ^{1}H NMR spectrum of **7a**.
Figure S18. ^{13}C NMR spectrum of **7a**.

Figure S1. HPLC of 60% CH_3CN extracts of Polygonaceous plants.

Reynoutria japonica

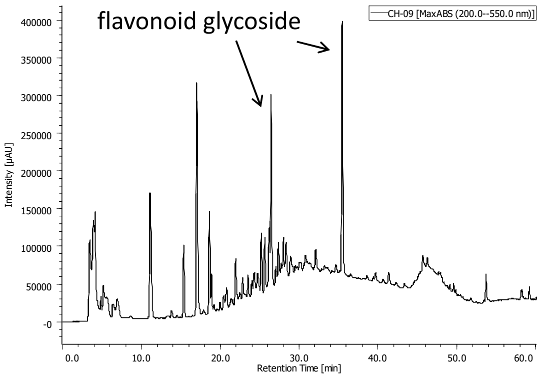

Persicaria perfoliata

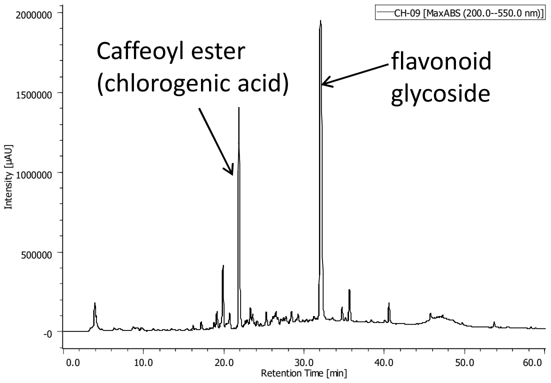

Persicaria longiseta

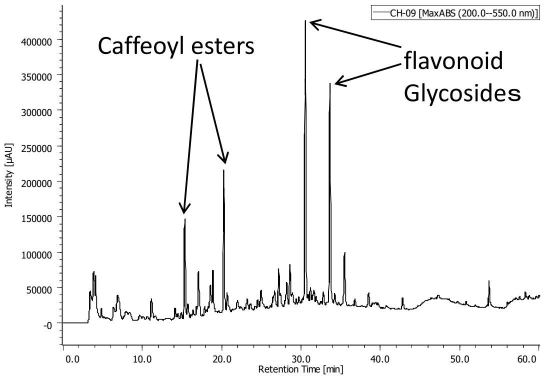

Persicaria lapathifolia

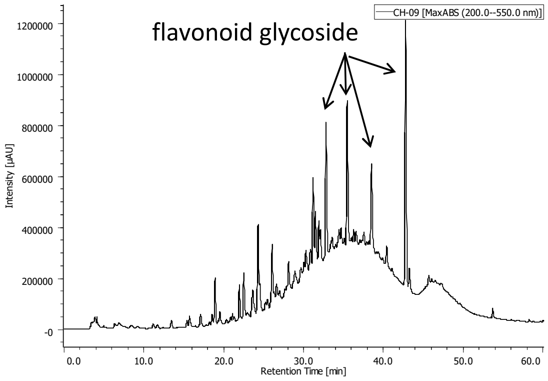

Persicaria filiformis

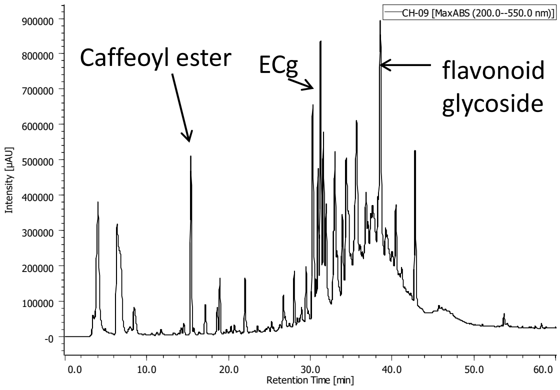

Persicaria thunbergii

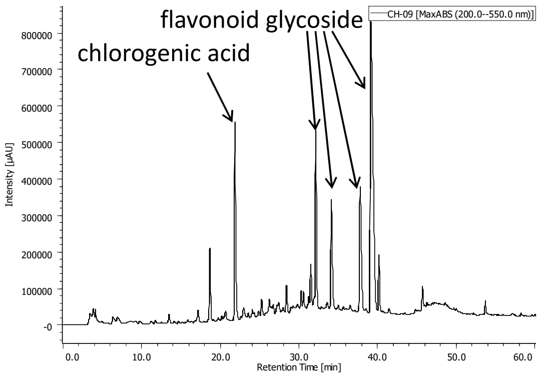

Figure S2. HPLC analysis of acid hydrolysis products of **3**.

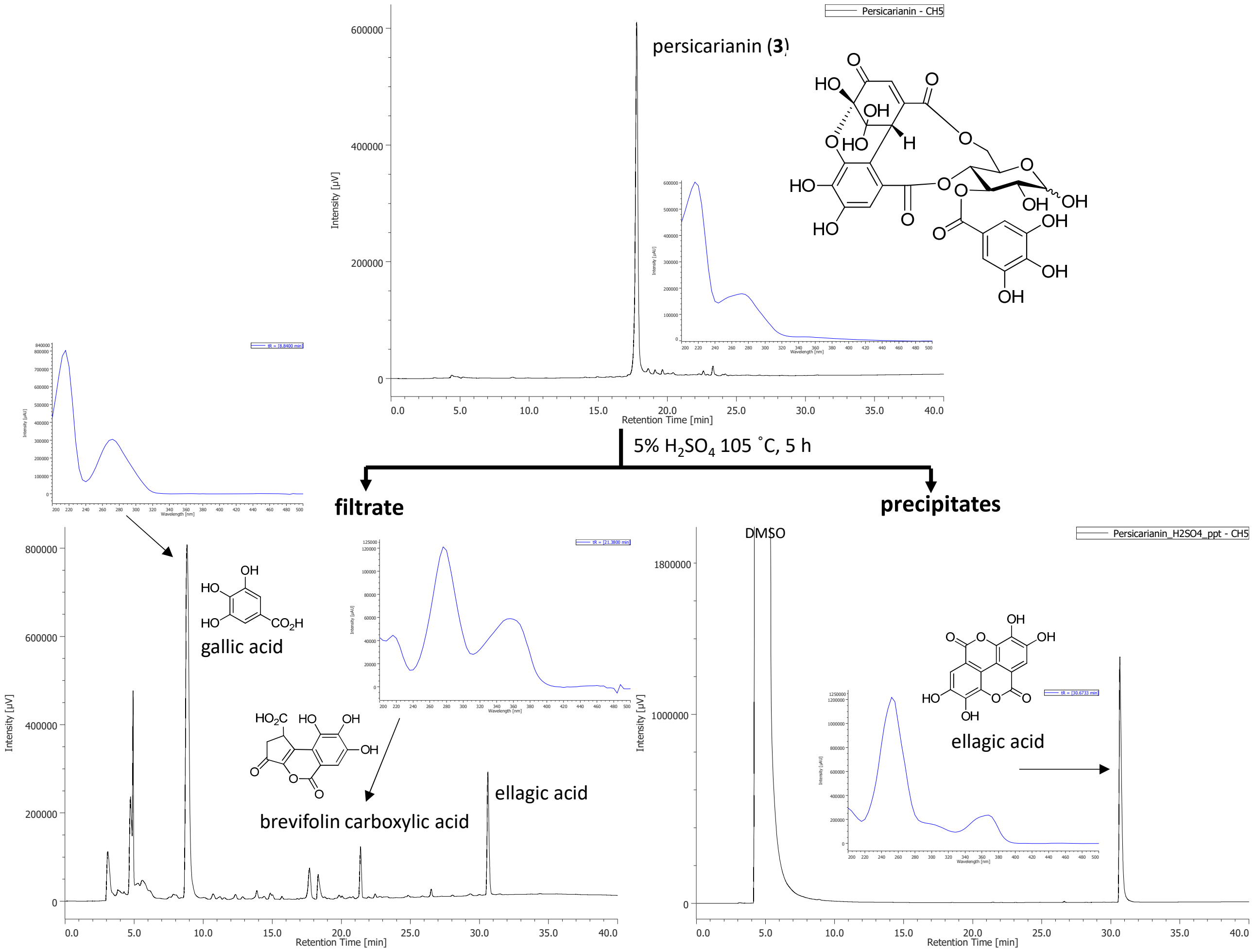

Figure S3. Thiol degradation of proanthocyanidin oligomers.

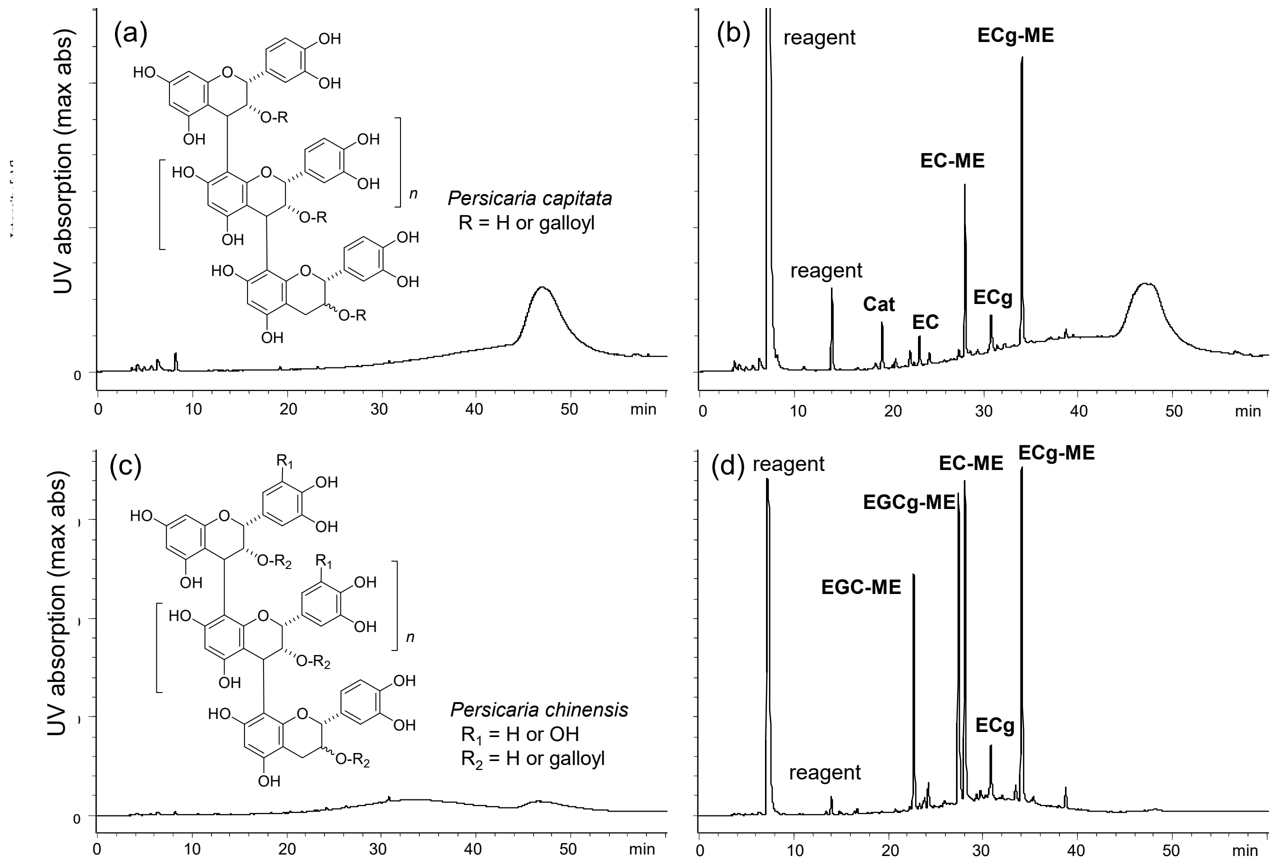

(a) Proanthocyanidin oligomers of *Persicaria capitata*, (b) Thiol degradation products of Proanthocyanidin oligomers of *Persicaria capitata*, (c) Proanthocyanidin oligomers of *Persicaria chinensis*, (d) Thiol degradation products of Proanthocyanidin oligomers of *Persicaria chinensis*.

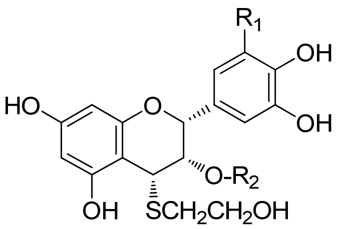

EC-ME : R_1 = H, R_2 = H
ECg-ME : R_1 = H, R_2 = galloyl
EGC-ME : R_1 = OH, R_2 = H
EGCg-ME : R_1 = OH, R_2 = galloyl

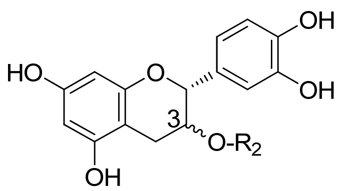

Cat : 3-β-OH
EC : 3-α-OH
ECg : 3-α-O-galloyl

Figure S4. ^{1}H NMR spectrum of **3**.

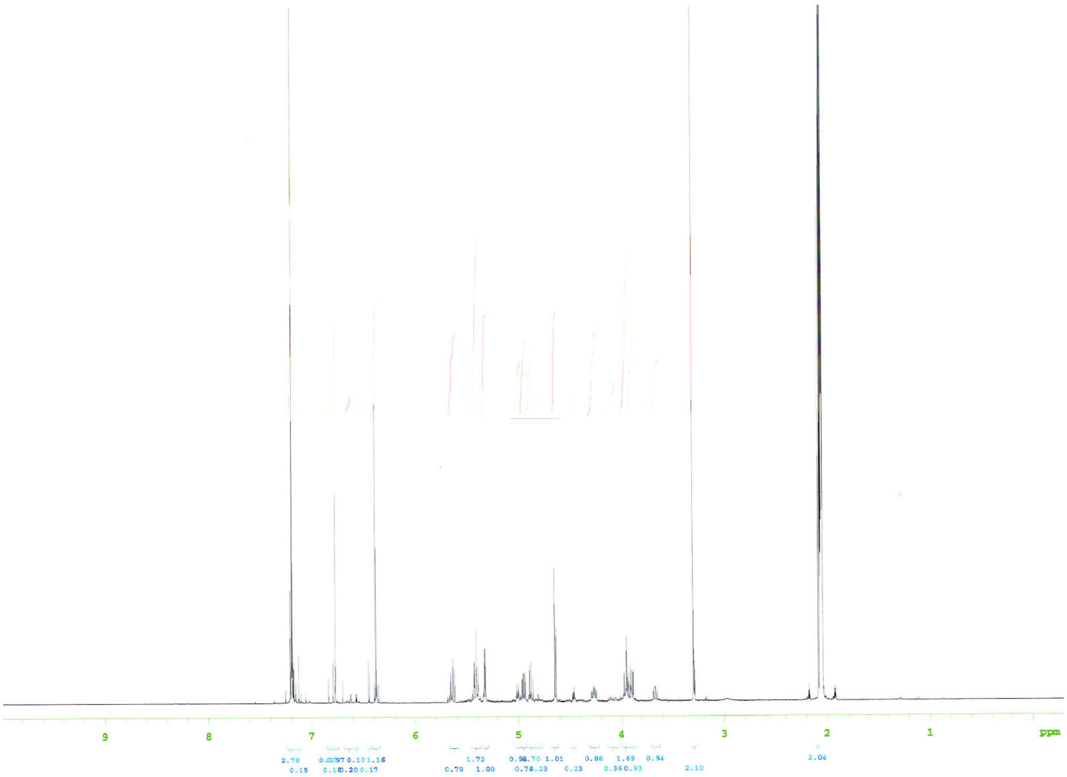

Figure S5. ^{13}C NMR spectrum of **3**.

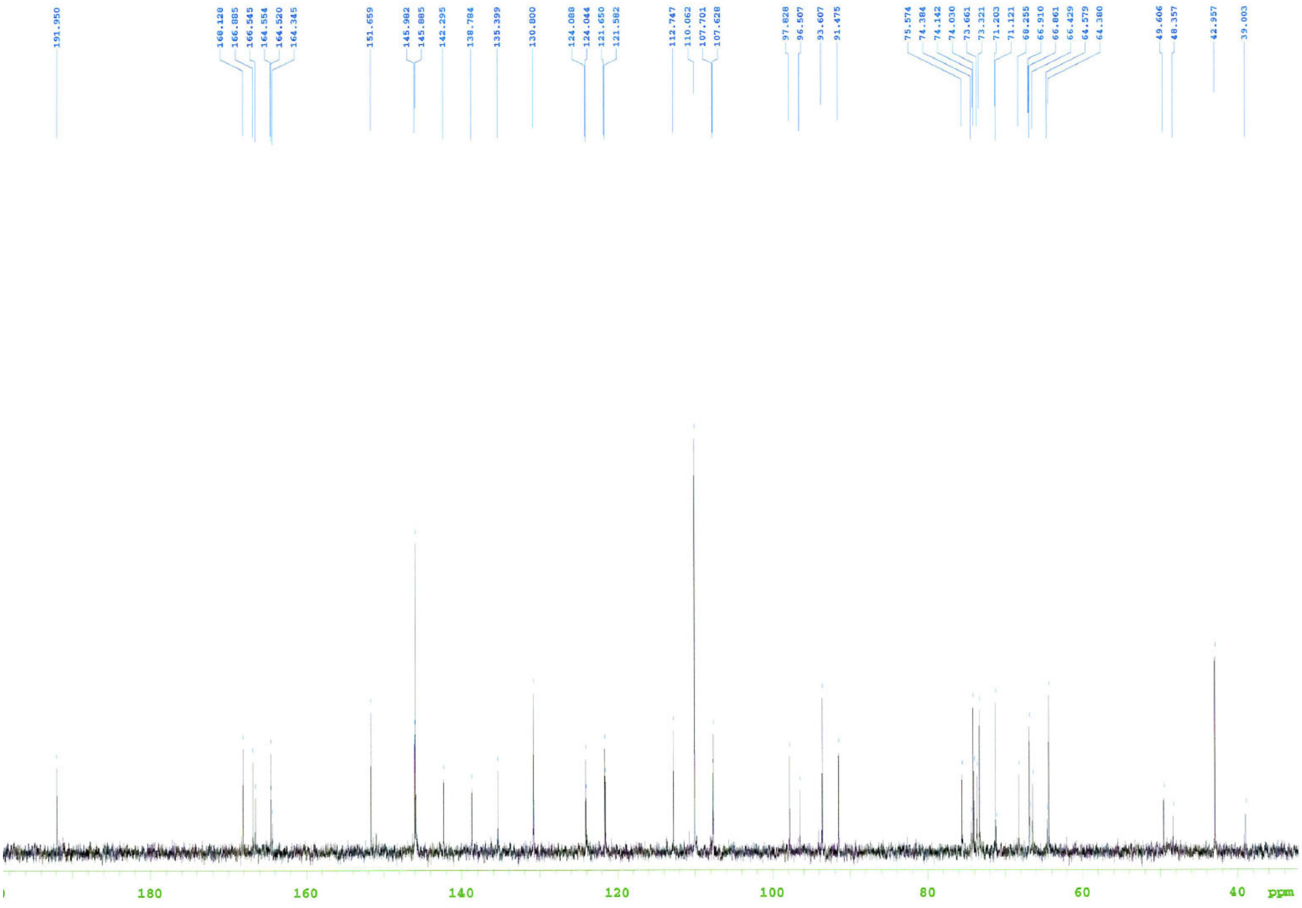

Figure S6. ^{1}H-^{1}H COSY spectrum of **3**.

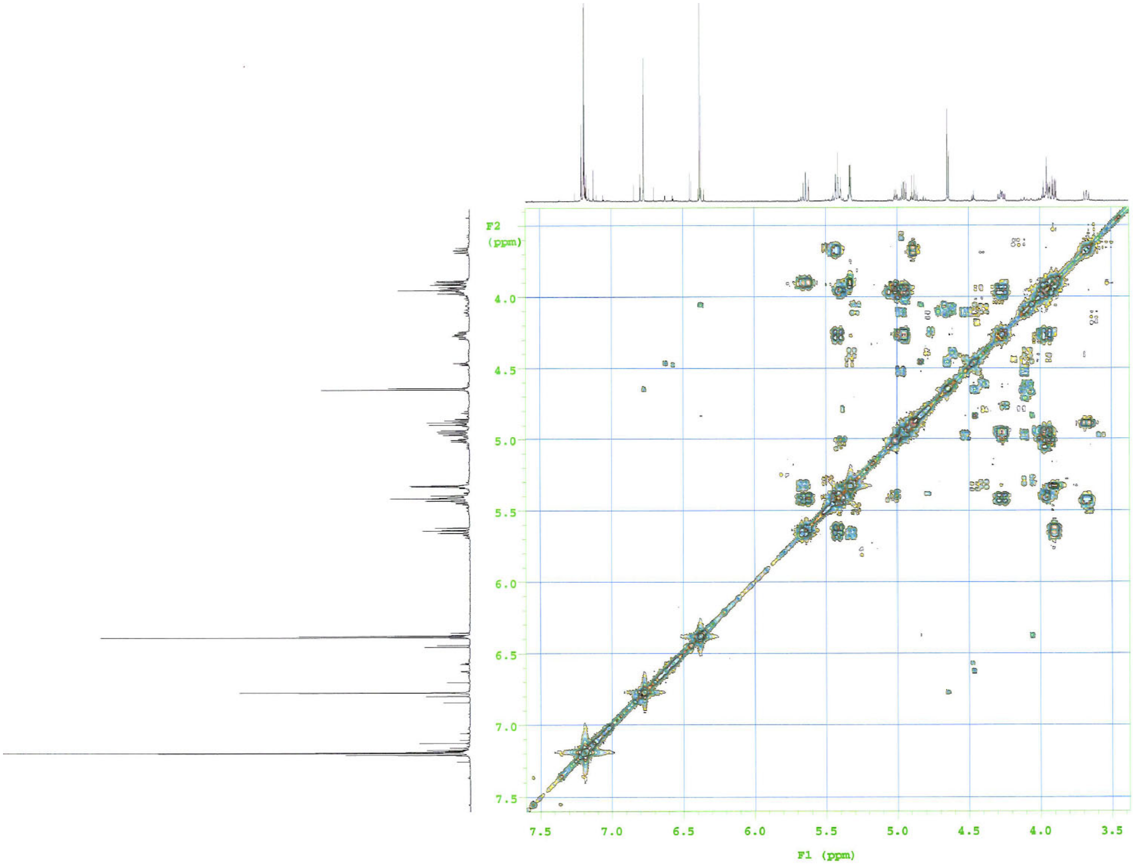

Figure S7. HSQC spectrum of **3**.

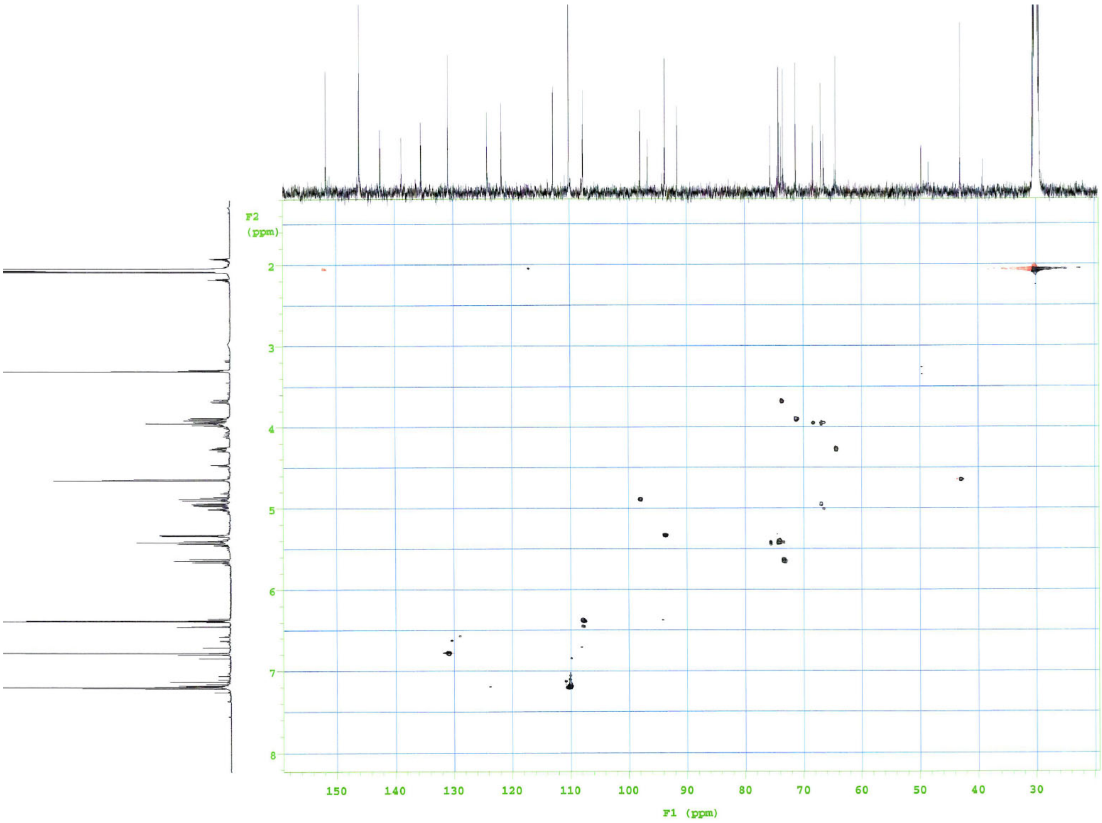

Figure S8. HMBC spectrum of **3**.

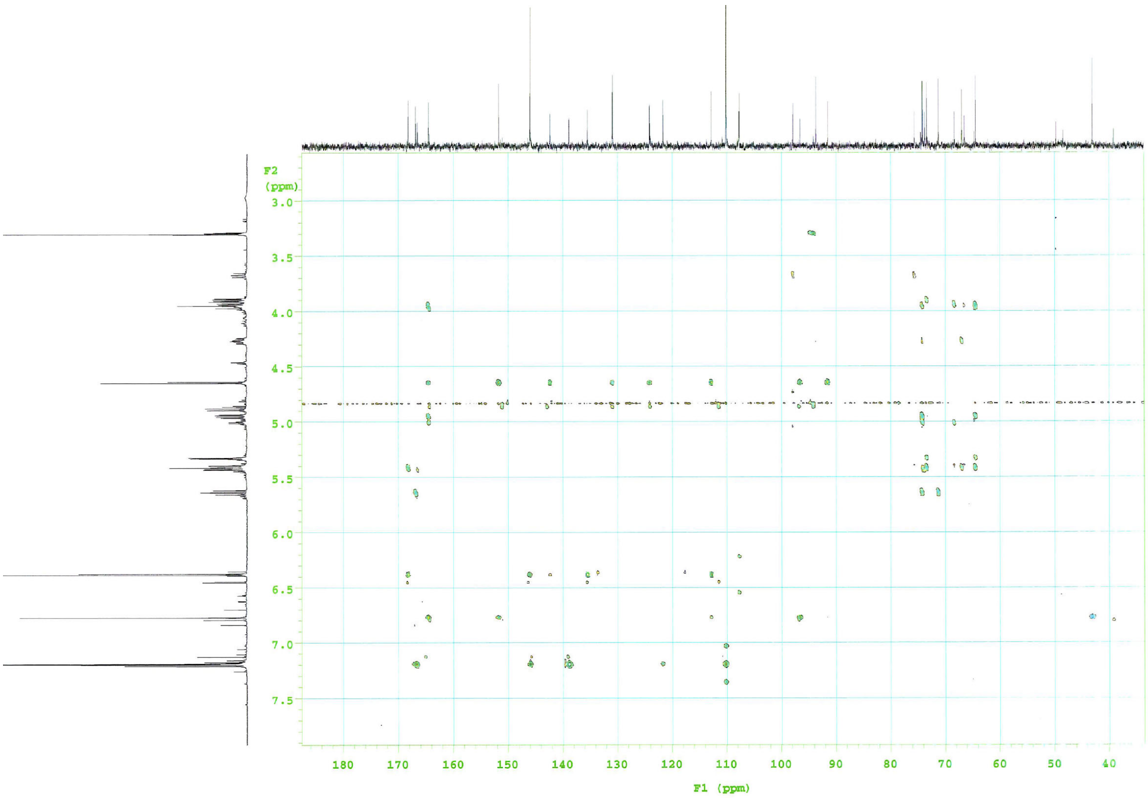

Figure S9. ^{1}H NMR spectrum of **11**.

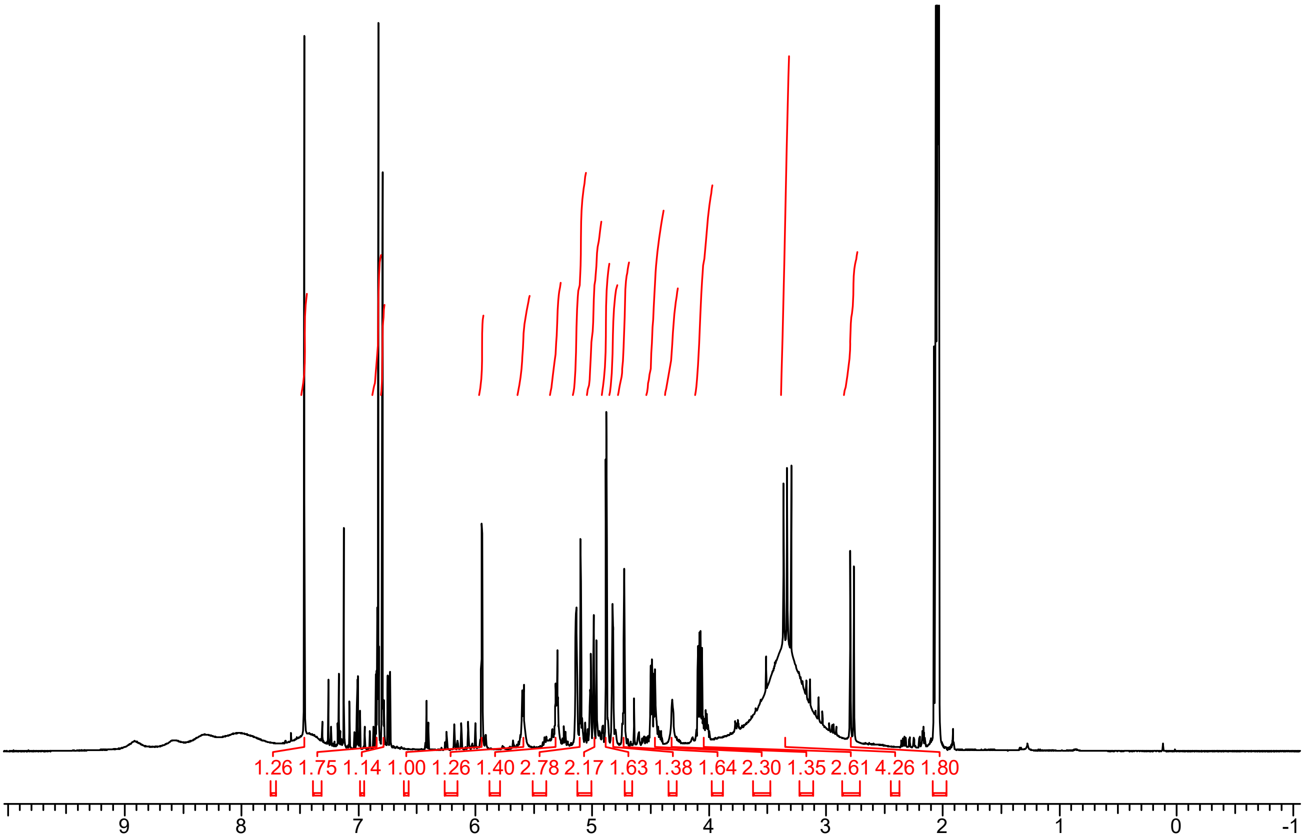

Figure S10. ^{13}C NMR spectrum of **11**.

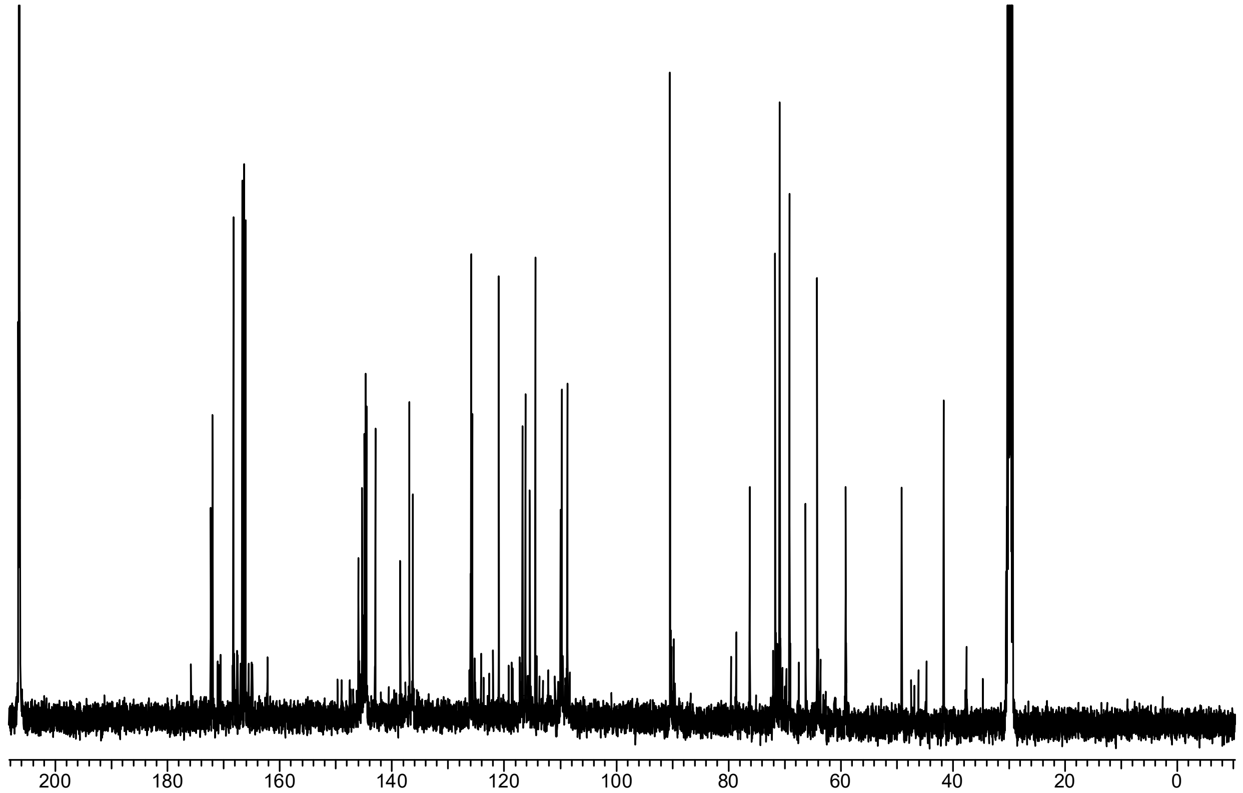

Figure S11. ^{1}H-^{1}H COSY spectrum of **11**.

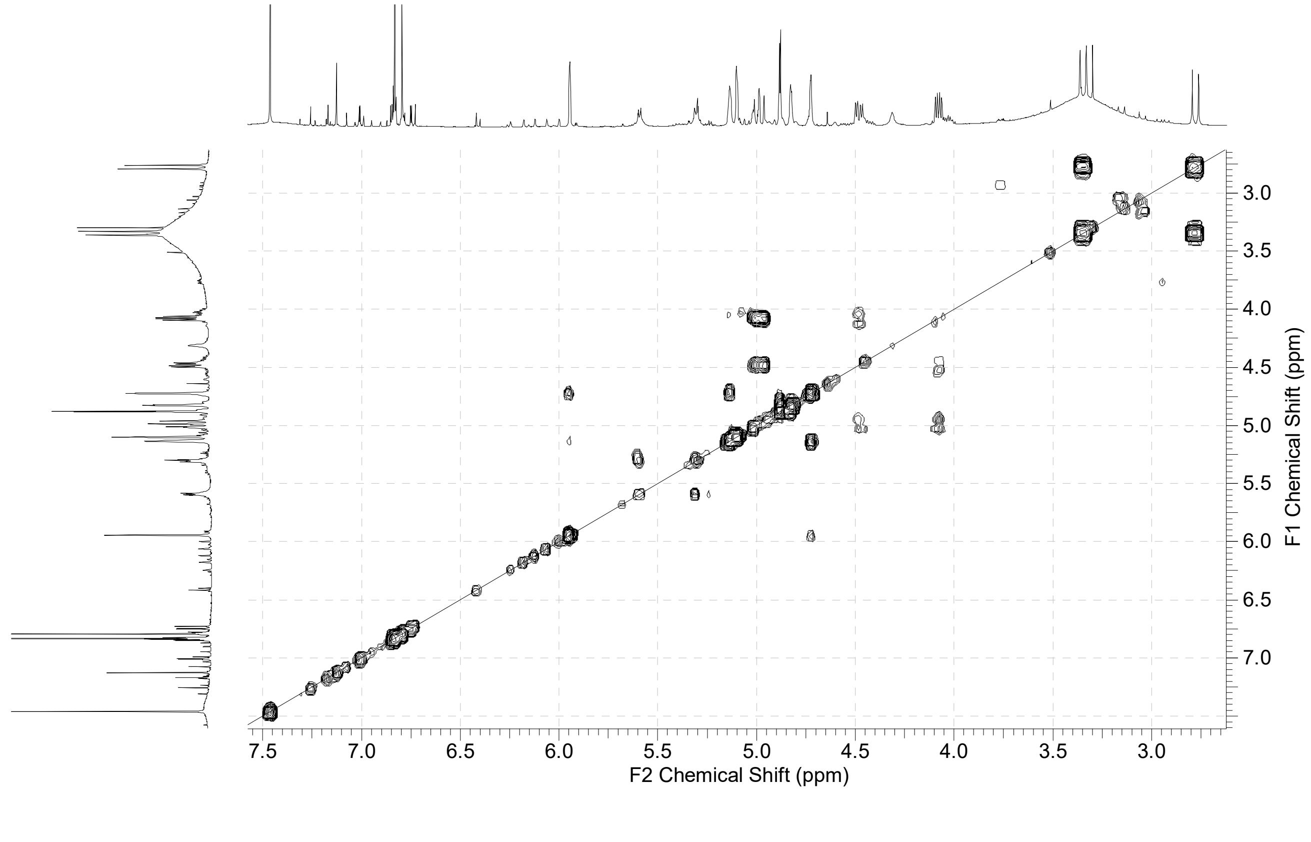

Figure S12. HSQC spectrum of **11**.

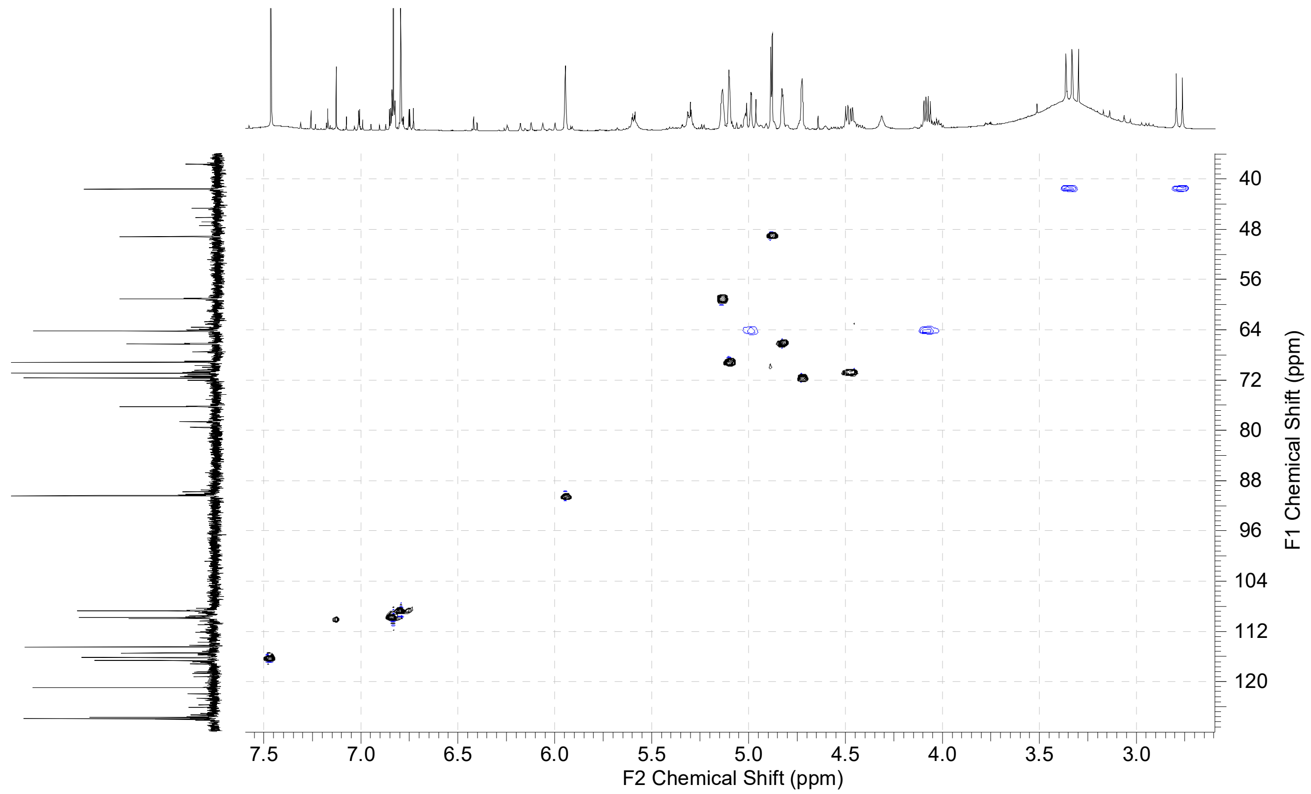

Figure S13. HMBC spectrum of **11**.

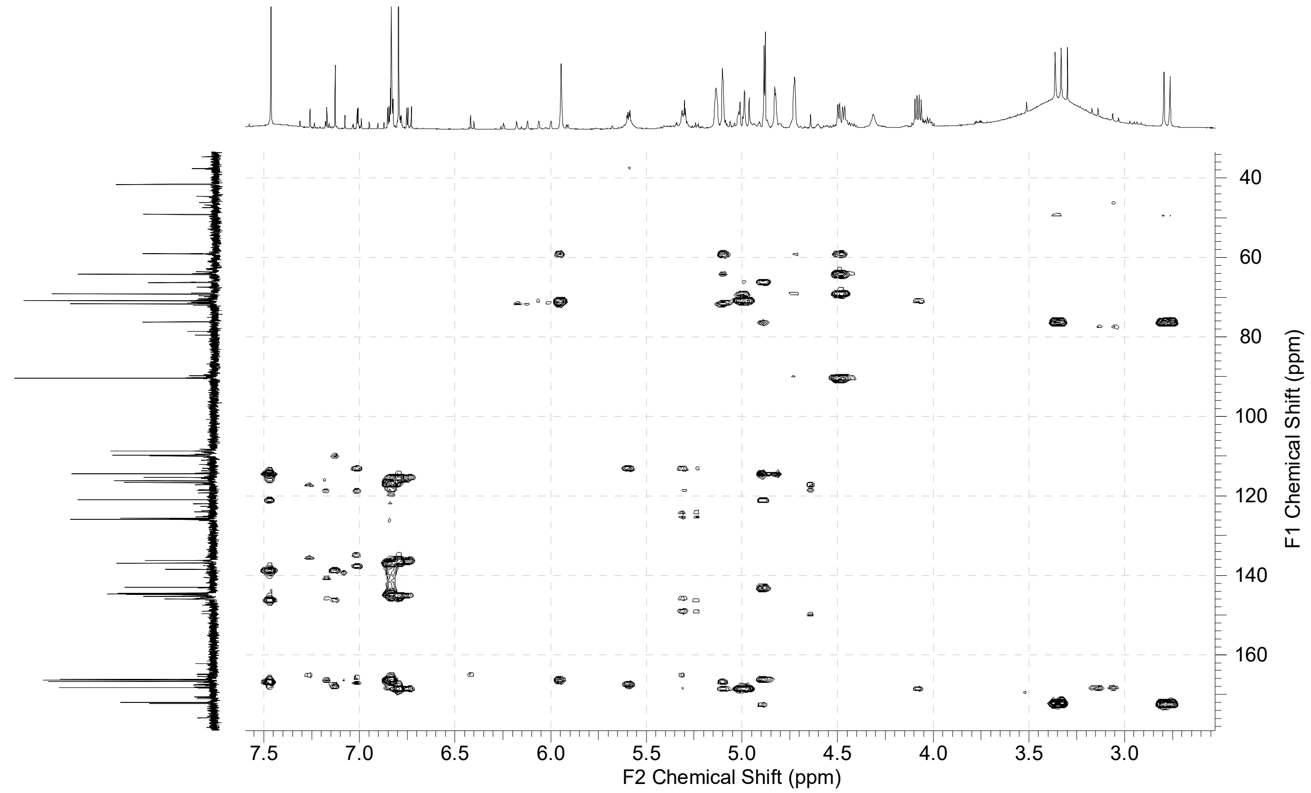

Figure S14. NOESY spectrum of **11**.

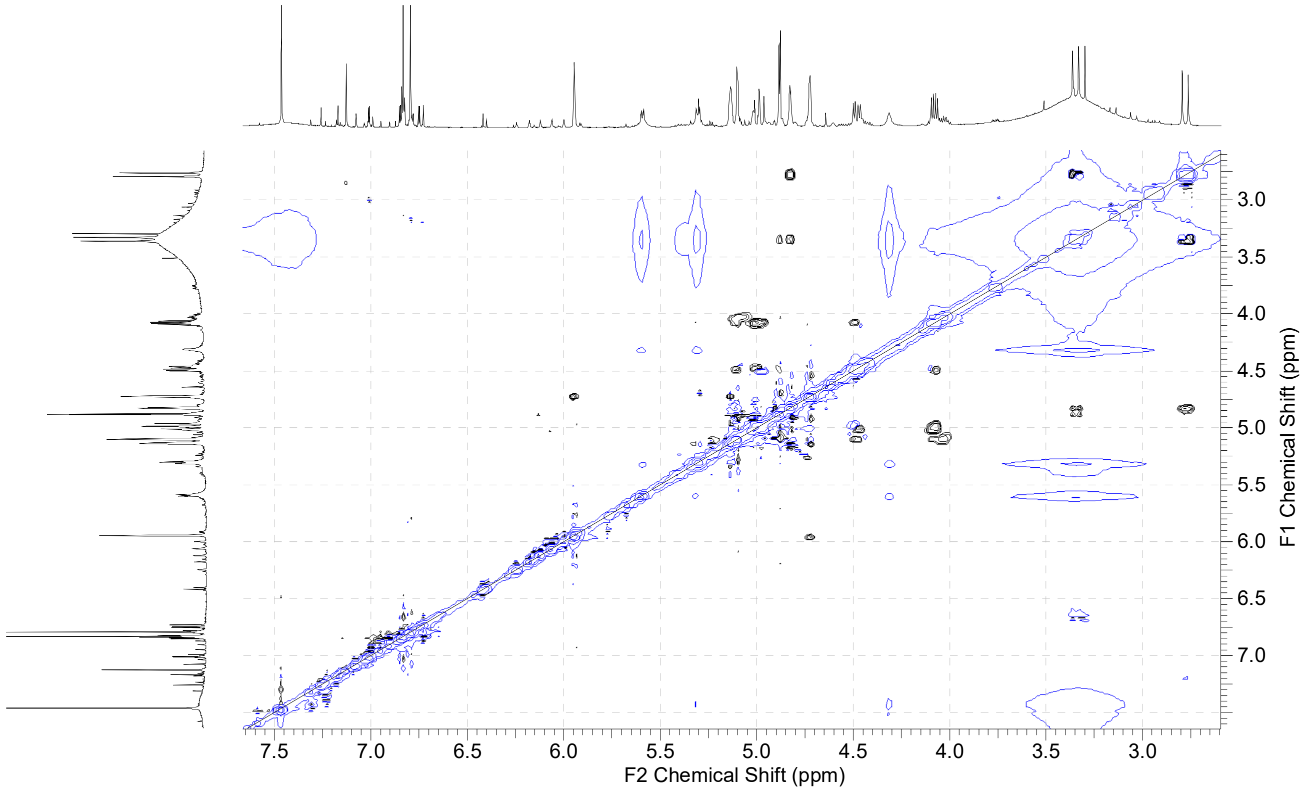

Figure S15. ^{1}H NMR spectrum of **7**.

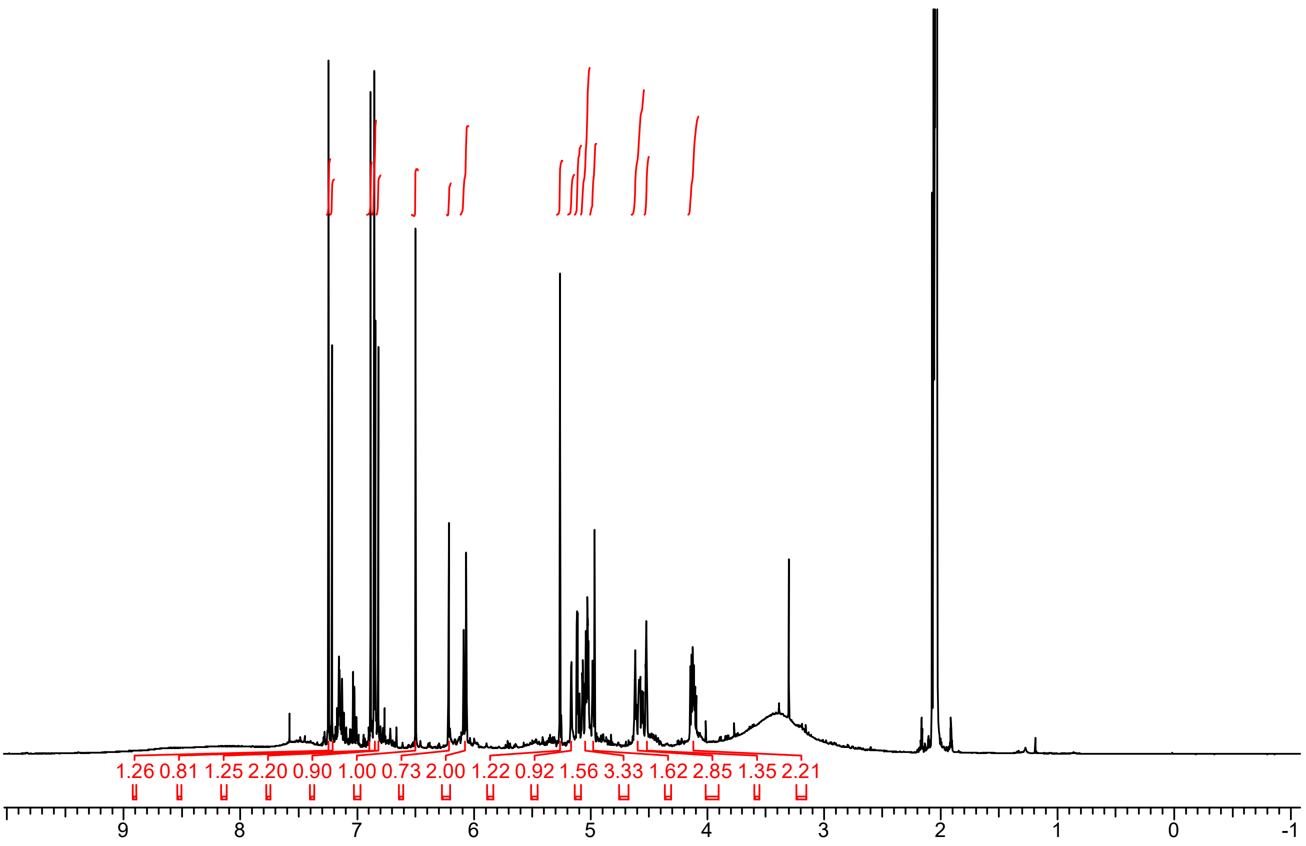

Figure S16. ^{13}C NMR spectrum of **7**.

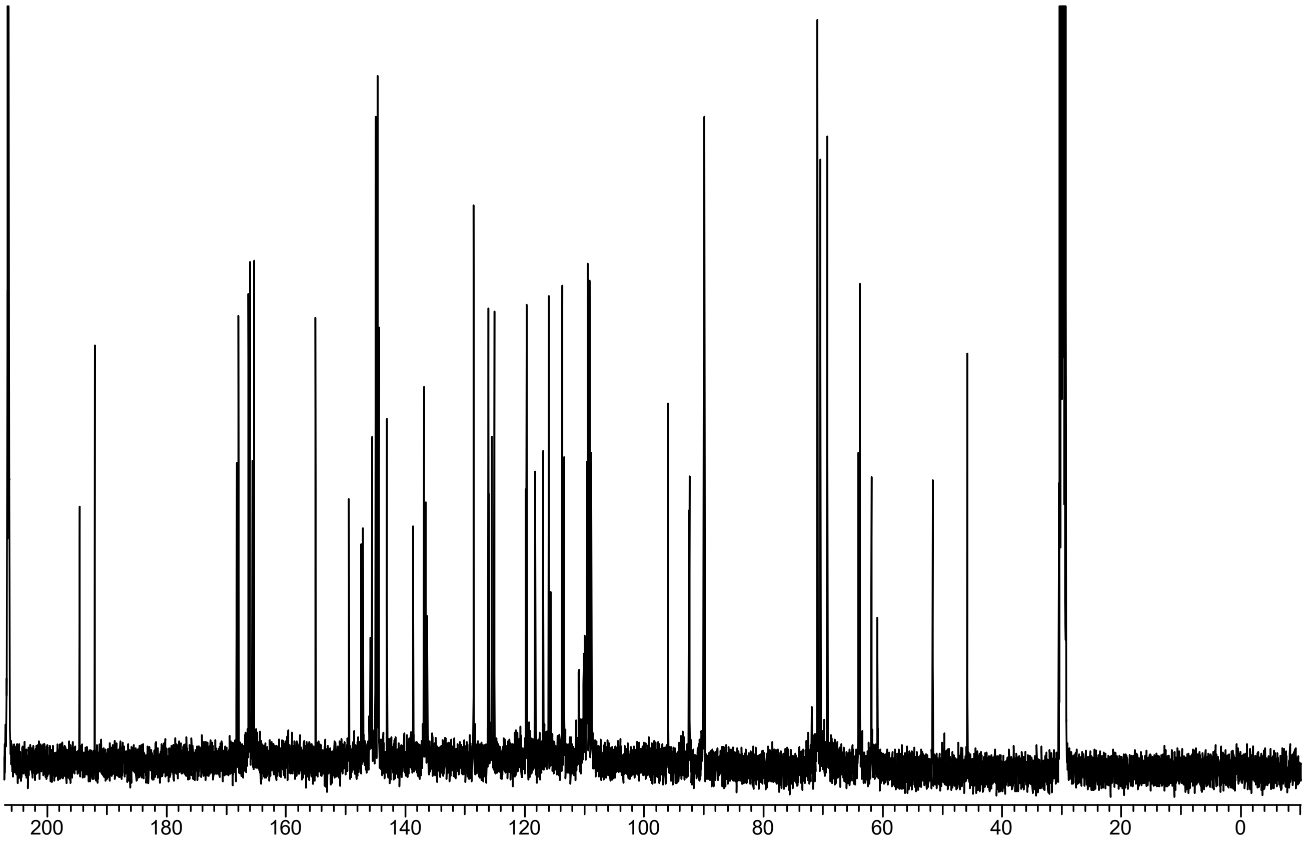

Figure S17. ^{1}H NMR spectrum of **7a**.

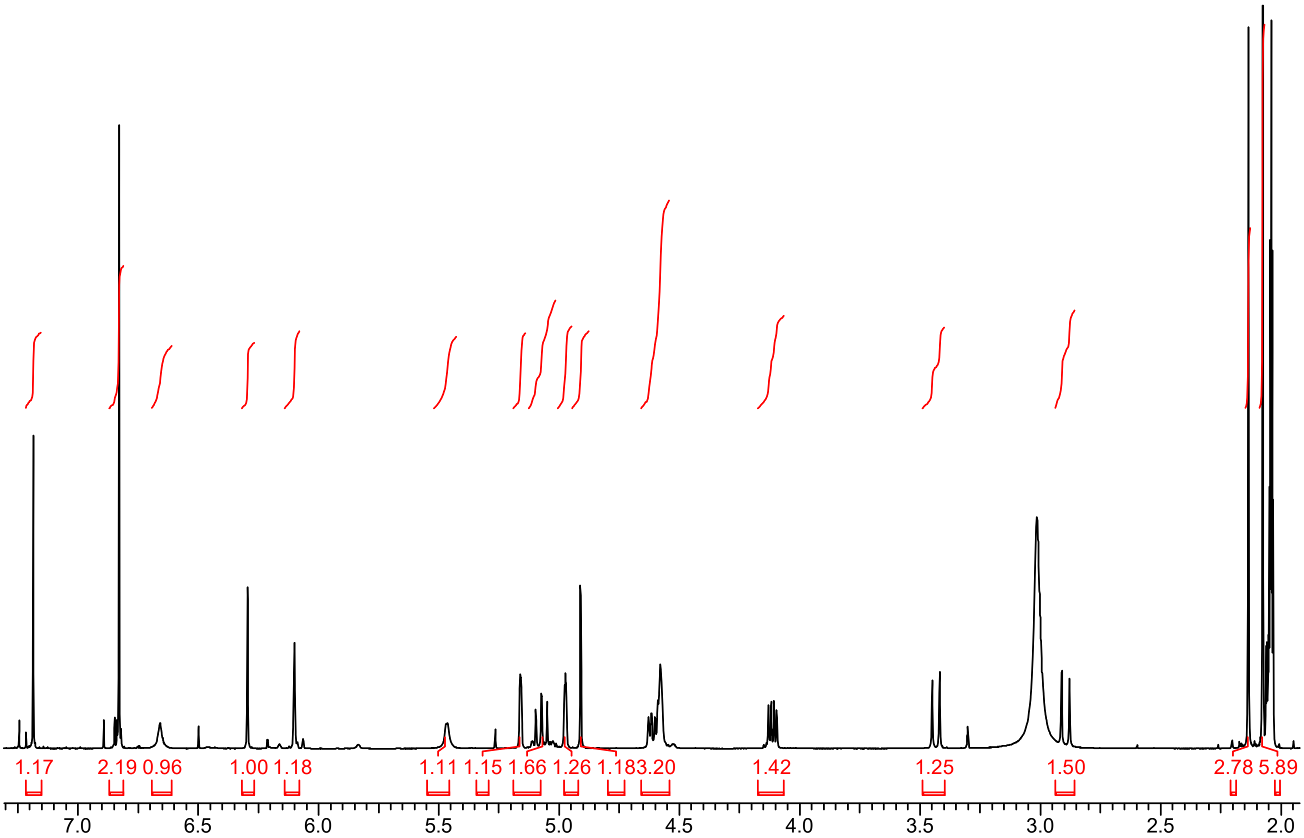

Figure S18. ^{13}C NMR spectrum of **7a**.

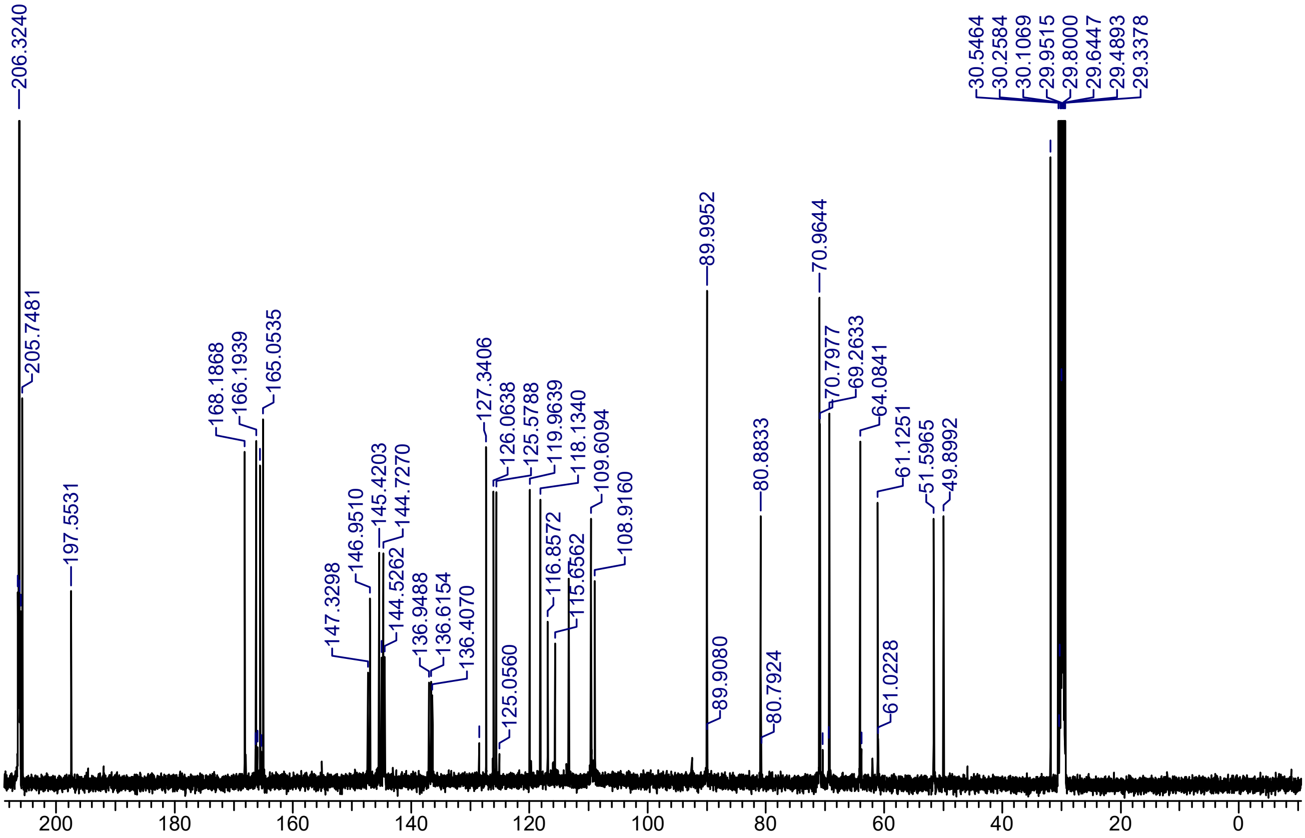

Supplement: Supplementary file 1 [file molecules-26-00337-s001.pdf]
